# Idiopathic cervical dystonia and non-motor symptoms: a pilot case-control study on autonomic nervous system

**DOI:** 10.1007/s10072-023-07033-y

**Published:** 2023-08-30

**Authors:** Fabiana Colucci, Maura Pugliatti, Ilaria Casetta, Jay Guido Capone, Enrica Diozzi, Mariachiara Sensi, Valeria Tugnoli

**Affiliations:** 1 Department of Neuroscience and Rehabilitation, University of Ferrara, Via Aldo Moro, 8, 44100 Ferrara, Italy; 2 Department of Clinical Neurosciences, Parkinson and Movement Disorders Unit, Fondazione IRCCS Istituto Neurologico Carlo Besta, Milan, Italy; 3 Department of Neuroscience and Rehabilitation, Azienda Ospedaliero-Universitaria S, Anna, Ferrara, Italy

**Keywords:** Cervical dystonia, Autonomic nervous systems, Laser Doppler flowmetry, Spectral analysis, COMPASS-31

## Abstract

**Purpose:**

Non-motor symptoms, such as sleep disturbances, fatigue, neuropsychiatric manifestations, cognitive impairment, and sensory abnormalities, have been widely reported in patients with idiopathic cervical dystonia (ICD). This study aimed to clarify the autonomic nervous system (ANS) involvement in ICD patients, which is still unclear in the literature.

**Methods:**

We conducted a pilot case-control study to investigate ANS in twenty ICD patients and twenty age-sex-matched controls. The Composite Autonomic System Scale 31 was used for ANS clinical assessment. The laser Doppler flowmetry quantitative spectral analysis, applied to the skin and recorded from indices, was used to measure at rest, after a parasympathetic activation (six deep breathing) and two sympathetic stimuli (isometric handgrip and mental calculation), the power of high-frequency and low-frequency oscillations, and the low-frequency/high-frequency ratio.

**Results:**

ICD patients manifested higher clinical dysautonomic symptoms than controls (*p* < 0.05). At rest, a lower high-frequency power band was detected among ICD patients than controls, reaching a statistically significant difference in the age group of ≥ 57-year-olds (*p* < 0.05). In the latter age group, ICD patients showed a lower low-frequency/high-frequency ratio than controls at rest (*p* < 0.05) and after mental calculation (*p* < 0.05). Regardless of age, during handgrip, ICD patients showed (i) lower low-frequency/high-frequency ratio (*p* < 0.05), (ii) similar increase of the low-frequency oscillatory component compared to controls, and (iii) stable high-frequency oscillatory component, which conversely decreased in controls. No differences between the two groups were detected during deep breathing.

**Conclusion:**

ICD patients showed ANS dysfunction at clinical and neurophysiological levels, reflecting an abnormal parasympathetic-sympathetic interaction likely related to abnormal neck posture and neurotransmitter alterations.

## Introduction

Cervical dystonia is a rare hyperkinetic movement disorder characterized by sustained, intermittent contractions of neck muscle leading to abnormal, often repetitive, postures, or both [1]. Idiopathic cervical dystonia (ICD) is the most common type of focal dystonia [2]; however, the overall prevalence of the disease is difficult to determine due to the heterogeneous methodology used across the studies [3]. All studies consistently report that ICD occurs twofold in women than in men, with a mean age of onset in the fifth and sixth decades, respectively [4–6].

At the pathophysiology level, it is hypothesized that dysfunctions in the cortico-striate-thalamus-cortical network and the cerebellum-thalamus-cortical pathway are involved in the development of cervical dystonia. Indeed, Stamelou et al. hypothesized that genetic susceptibility might predispose to the neurochemical and functional imbalance in the basal ganglia (i.e., lower concentrations of GABA and dopamine and higher levels of acetylcholine), leading to brain networks abnormalities [7], linked to both the motor [8] and non-motor processes [7]. Currently, interest in non-motor symptoms associated with ICD has increased. Neuropsychiatric symptoms (depression and anxiety) [9], sleep disturbances [10, 11], fatigue and cognitive impairment [12, 13], sexual dysfunction [14], and sensory abnormalities [15, 16] have been widely reported in ICD patients. Conversely, the autonomic nervous system (ANS) involvement in this group of patients is still controversial and unclear.

The ANS dysfunction is widely studied and demonstrated in hypokinetic movement disorders, mainly Parkinson’s disease and multiple system atrophy [17]. However, ANS might be involved in different movement disorders. The first description of ANS in the context of cervical dystonia patients was reported by Tiple et al., who detected in ICD patients a parasympathetic involvement in the spectral analysis of heart rate variability [18].

In addition, Hentschel and colleague [19] quantitatively investigated ANS in patients with ICD and depression with the clinical questionnaire modified from Low et al., detecting hypotension and constipation. Since that time, studies on ANS involvement in ICD patients have been very few [19].

An easier and quicker self-rating questionnaire, reported as a valuable tool for detecting ANS involvement in several neurological diseases, is the Composite Autonomic Symptoms Scale 31 (COMPASS-31).

To our knowledge, studies on quantitative spectral analysis of laser Doppler flowmetry (LDF) applied to the skin to assess both ANS components (sympathetic and parasympathetic) have not yet been reported in patients with ICD, nor have studies on ANS from the clinical point of view through the Composite Autonomic Symptoms Scale 31 (COMPASS-31).

Therefore, the purpose of the present pilot study was to compare ANS function in ICD patients versus controls by clinical and neurophysiological tests: COMPASS-31 and quantitative spectral analysis of LDF. Second, we sought to highlight the correlations between ANS function and cervical dystonia severity in ICD patients.

## Methods

We conducted a case-control pilot study to investigate the autonomic nervous system function in patients with idiopathic cervical dystonia versus control. Cases were selected among those treated with botulinum toxin at the Movement Disorder Centre of the Ferrara Hospital.

### Subjects

This is a monocentric study conducted at the Movement Disorder Centre of Ferrara Hospital. Participants gave informed content, and the study protocol received approval from the local ethical committee (ID 693-111- 835/2020/Oss/AOUFe).

We selected a consecutive series of idiopathic cervical dystonia subjects who underwent neurological clinical assessment between May 2020 and August 2020 who fulfilled the following criteria:

- Age ≥ 18 years old.
- Diagnosis of idiopathic cervical dystonia according to phenomenology and neurophysiological data [1, 20].
- Chronic botulinum toxin treatment with the last performed at least twelve weeks earlier.
- Absence of main pathogenic gene variants known to be linked with dystonia (DYT1 and DYT6).
- Absence of history of drug-induced dystonia.
- Absence of deep brain stimulation (DBS) implant.
- Absence of hypertension and diabetes.
- Absence of dementia and/or other movement disorder diseases (combined dystonia).
- If on chronic antidepressant treatment and GABAergic/antimuscarinic/antiadrenergic agents, they should be taken more than 48 hours previously.

Patients were age-sex-matched with healthy male and female volunteers, not suffering from hypertension, diabetes, dementia, and/or other movement disorder diseases, and if on chronic antidepressant treatment and GABAergic/antimuscarinic/antiadrenergic agents, they should be taken more than 48 h previously.

Smoking was prohibited in all subjects before the evaluation.

### Clinical data

Physiological, family, and medical history was obtained for each subject. Data included demographics and lifestyle information, family neurological history (with emphasis on dystonia and/or depression), comorbidities, and medications.

All subjects completed the Composite Autonomic Symptoms Scale 31 (COMPASS-31) to measure global autonomic symptoms. The scale assesses through 31 questions autonomic symptoms in multiple domains [21]: orthostatic intolerance, vasomotor, secretomotor, gastrointestinal, bladder, and pupillomotor. In addition, subjects completed the Beck Depression Inventory (BDI) scale for assessing depressive symptoms.

The cases provided additional information on the age at onset of ICD, disease duration, and botulinum toxin treatment details. ICD patients were clinically assessed with the Toronto Western Spasmodic Torticollis Rating Scale (TWSTRS) and the Global Dystonia Severity Rating Scale (GDS).

### Neurophysiological data

ANS was assessed by quantitative spectral analysis of laser Doppler flowmetry (LDF) applied to the skin. In quantitative spectral analysis, any stable and fluctuating signal is decomposed into its sinusoidal components, allowing the power of each component and its respective frequency to be calculated. During deep breathing, handgrip, and mental arithmetic calculation, changes in heart rate modify the spectral skin-LDF recording and provide information on ANS activity. Deep respiratory activity increases the high-frequency (HF) spectral band [22], which reflects the vagal tone [23], whereas handgrip and mental arithmetic calculation act on the low-frequency (LH) spectral band [24, 25], reflecting the sympathetic tone [26].

Measurements were obtained after 4 h of abstinence from smoking and 48 h without taking medication that affects vascular tone. During the local heating test, participants sat in an armchair with neck support to permit the relaxation of the dystonic component in ICD patients. We applied thermostatic laser Doppler probes to both indices and set the local heating to 37 °C. Once the required temperature was reached, the cutaneous laser Doppler flowmetry was applied simultaneously at rest for 2 min, after six deep breaths (5-s-long expiration and inspiration, respectively), after an arithmetic calculation (subtracting three from 200, again three from the resulting number, and so on for 2 min), and after isometric handgrip (40% of maximum force with the dominant hand lasting 2 min, recording from the contralateral). Subjects waited 2 min to reassess the normal ANS status between each condition. We analyzed right and left indices to investigate whether the side of the torticollis might influence the recording in the ICD group.

We obtained short-term power spectra recordings with the Perimed software. We considered two frequency bands: high-frequency (HF) power fluctuations (0.081 to 1.5), reflecting vagal activity, and low-frequency (LF) power fluctuations (0.003 to 0.080), reflecting sympathetic modulation [27]. The Perimed software detected four main frequency bands. We calculated both HF and LF total power by summing the power of each frequency obtained according to the previously described values. Then, we computed the LF/HF ratio during all the conditions (rest, deep breathing, handgrip, and mental calculation).

### Statistic analysis

Results are expressed as counts and percentages for categorical variables and mean and standard deviations (SD) for continuous variables. The distributions of categorical variables among groups of individuals were analyzed with the chi-square test, whereas the distribution of continuous variables was evaluated with the Shapiro-Walk normality test. To compare clinical and instrumental data distribution between the two groups, the means of normally distributed continuous variables were assessed with Student’s t-test, and for continuous variables with non-normal distribution, differences were assessed with Mann-Whitney’s U-test. A *p*-value of < 0.05 indicated a statistical significance.

Although the sample size is small, we performed a secondary analysis among participants aged 57 years old and older to correct for age in assessing ANS changes. Indeed, the aging process has been associated with structural and functional changes in the autonomic nerve and ganglia, leading to sympathetic tone predominance and vagal tone reduction [28]. Therefore, we would like to evaluate if this bias could interfere with the statistical analysis of the initial groups. To choose the cut-off, knowing that there is a linear correlation between age and ANS changes, we choose patients with age at enrollment higher than the mean age of the participants groups.

Statistical analyses were performed with IBM Statistical Package for Social Science (SPSS).

## Results

We enrolled twenty ICD patients and twenty age- and sex-matched healthy subjects (mean age 56.9 ± 11.1 years old). No demographic, family history, or lifestyle differences were found between cases and controls (Table 1).

**Table 1:** Demographical and clinical data in cervical dystonia patients and control subjects

	Cases (*N* = 20), mean ± DS	Controls (*N* = 20), mean ± DS	*p-*value
Sex (F/M)	12/8	12/8	ns
Mean age (years)	56.9 (11.1)	56.9 (11.1)	ns
Female	56 (8.8)	56 (8.8)	ns
Male	58.3 (13.8)	58.3 (13.8)	ns
Smokers (number)	3	6	ns
Female	2	4	ns
Male	1	2	ns
Coffee intake (number of subjects)	13	16	ns
Female	6	10	ns
Male	7	6	ns
Alcohol use (number of subjects)	13	17	ns
Female	7	9	ns
Male	6	8	ns
Positive familiar history of dystonia and/or depression and/or dementia (number of subjects)	5	7	ns
Depression	2	1	ns
Dementia	2	3	ns
Depression + dementia	1	3	ns
Dystonia	0	0	ns
Other neuro-psychiatric diseases	0	0	ns
COMPASS-31 (number of subjects with at least one domain declared)	17	7	*p* **< 0.05**
Orthostatic intolerance	7	1	*p* **< 0.05**
Vasomotor	0	0	ns
Secretomotor	5	2	ns
Gastrointestinal	11	4	*p* **< 0.05**
Bladder	2	0	ns
Pupillomotor	0	0	ns
BDI	6.20 (4.53)	3.10 (1.74)	*p* **< 0.05**

*COMPASS-31* Composite Autonomic Symptoms Scale 31, *BDI* Back Depression Index

Significant values are bolded

Cases compared to controls showed at least one autonomic symptom on the COMPASS-31 scale (86% versus 14%; *p* < 0.05). The main COMPASS-31 domains affected were orthostatic intolerance (35% versus 5%) and gastrointestinal (55% versus 20%), less secretomotor (25% versus 10%), and bladder dysfunction (10% versus 0%); pupillomotor and/or vasomotor symptoms were not reported. In cases, patients with two or more domains involved on the COMPASS-31 scale showed greater ICD severity on TWRSTS than those with 0 or 1 domain affected (TWRSTS median: 25.5 (IQR 16.5–28) versus TWRSTS median: 15 (IQR 13.5–15); (*p* < 0.05)). No other differences were detected for the additional clinical data between females and males (Table 2).

**Table 2:** Clinical dystonia data in gender and age at enrollment subgroups

	Cases (20)	Female (12)	Male (8)	*p*-value	Patients < 57 years old (13)	Patients > 57 years old (7)	*p*-value
Mean age at disease onset (media years—SD)	41.3 (11.6)	40.3 (10.7)	42.9 (13.5)	ns	35.08 (8.35)	52.86 (6.99)	ns
Disease duration (media/median years—SD/IQR)	15.7 (6.4)	15.5 (8.4)	15.8 (5.1)	ns	14.0 (9.0–20.0)	18.0 (12.5–20.5)	ns
Duration of botulinum toxin treatment (media/median years-SD/IQR)	12.3 (6.7)	12.4 (4.7)	12.2 (9.2)	ns	12.0 (8.0–15.0)	12 (10.0–16.0)	ns
TWSTRS (media—SD)	22.5 (8.3)	25.7 (8.4)	17.7 (5.2)	ns	23.4 (9.2)	20.7 (6.4)	ns
GDS (media—SD)	8.0 (2.1)	8.5 (2.1)	7.6 (2.1)	ns	8.4 (2.3)	7.9 (2.0)	ns
COMPASS-31 (number of patients with at least one domain)	16	10	6	ns	9	7	ns

*COMPASS-31* Composite Autonomic Symptoms Scale 31, *SD* standard deviation, *GDS* Global Disease Scale, *IQR* interquartile range, *TWSTRS* Toronto Western Spasmodic Torticollis Rating Scale

**Fig. 1:**
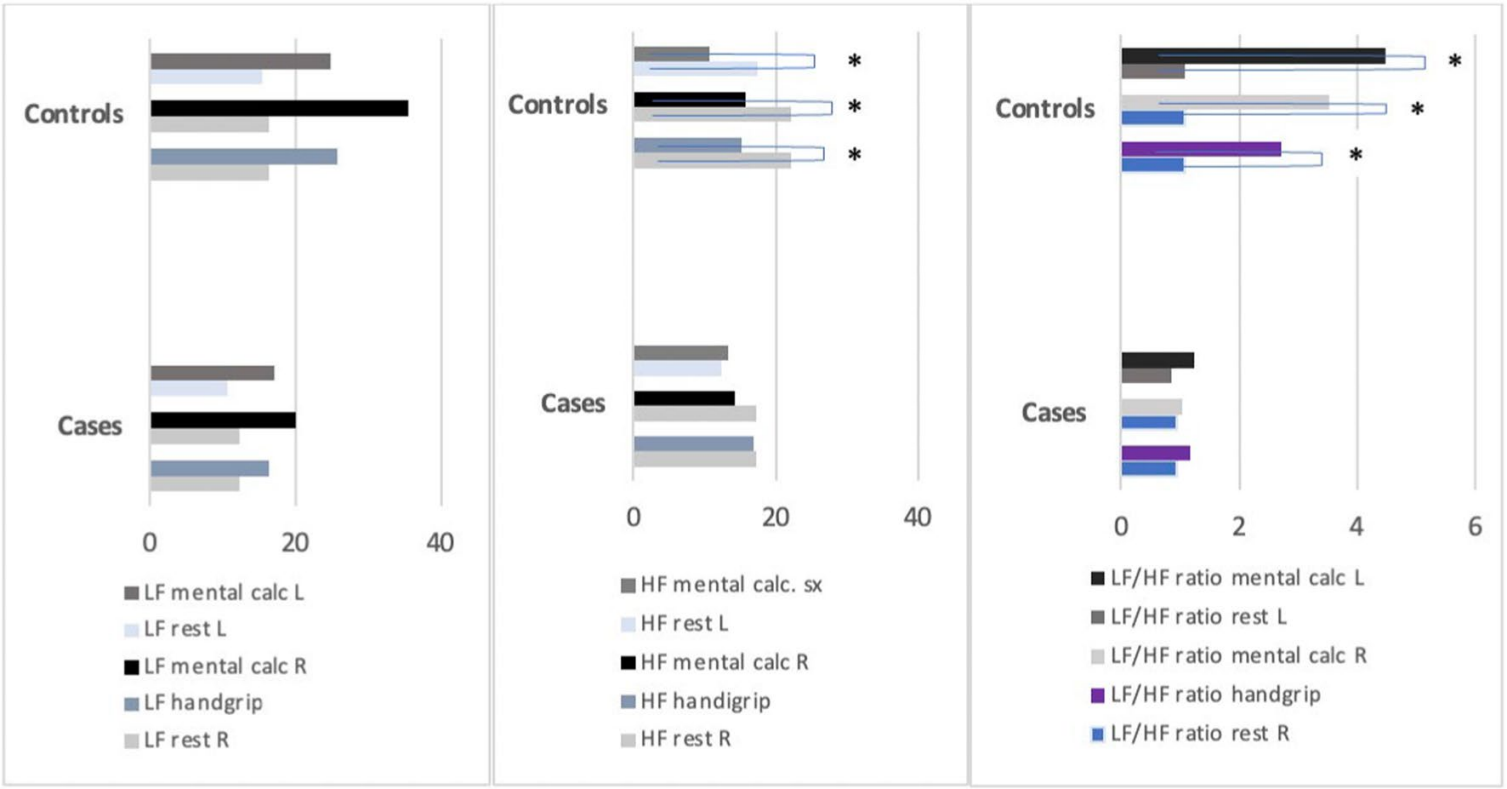
Graphic representation of low and high frequencies at rest and in orthosympathetic conditions. The graphic shows during orthosympathetic activation (handgrip and mental calculation), the increment and decrement of LF and HF, respectively. *HF* high frequencies, *L* left, *LF* low frequencies, *R* right. * indicates the significant values

LDF spectral analysis at rest, recording from the right (RI) and left indices (LI), showed lower high-frequency (HF) bands in cases compared to controls (median RI-HF: 17.20 versus 22.05, median LI-HF: 12.40 versus 17.40), although without reaching statistical significance. The same results were obtained by analyzing LF/HF ratio (median RI-LF/HF: 0.94 versus 1.07, median LI-LF/HF: 0.85 versus 1.07) (Fig. 1).

**Fig. 2:**
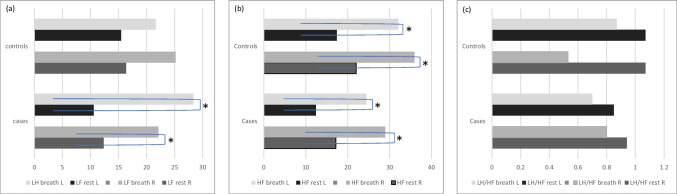
Graphic representation of low and high frequencies at rest and in parasympathetic condition. The graphic shows during parasympathetic activation, LF and HF changes. **a** Low frequencies changes, **b** High frequencies changes, **c** LF/HF changes. *HF* high frequencies, *L* left, *LF* low frequencies, *R* right. * indicates the significant values

Parasympathetic activation led to no LF/HF ratio differences (median RI-LF/HF: 0.80 versus 0.53, median LI-LF/HF: 0.70 versus 0.87), and all participants showed a significant increase in the HF spectral band from the rest condition (*p* < 0.05; 70% of cases and controls) (Fig. 2).

Any statistical difference in LF, HF, and LF/HF ratio were detected at the LDF spectral analysis during mental calculation (Table 3). In contrast, during handgrip, cases showed a lower LF/HF ratio than controls (median (IRQ) LF/HF: 1.17 (0.56–2.76) versus 2.72 (1.11–4.27)) (*p* < 0.05; 80%) (Table 3). During these sympathetic stimuli, the increase of LF power from the rest spectral band was similar in cases and controls, whereas the HF power significantly decreased only in the control group (*p* < 0.05) (Fig. 1). In addition, both groups showed an increase in the LF/HF ratio from rest to handgrip or mental calculation, reaching a statistically significant difference only in controls (*p* < 0.01 from rest to handgrip, *p* < 0.05 from rest to mental calculation) (Table 3).

**Table 3:** Laser Doppler flowmetry spectral analysis data

Condition	Cases	Controls	*p*-value	Case ≥ 57 yo	Controls ≥ 57 yo	*p*-value
Rest						
LH						
Right	12.35 (9.52–33.22)	16.35 (10.47–60.52)	ns	12.90 (11.40–28.50)	14.00 (9.85–22.05)	ns
Left	10.55 (6.82–29.85)	15.45 (9.07–21.40)	ns	10.10 (7.40–15.95)	18.6 (10.75–19.90)	ns
HF						
Right	17.20 (10.50–34.62)	22.05 (17.62–25.57)	ns	11.25 (20.90–39.00)	21.40 (17.30–23.40)	***p*** **< 0.05**
Left		17.40 (7.85–23.75)	ns	10.2 (8.35–30.70)	19.00 (17.10–22.60)	***p*** **< 0.05**
LF/HF ratio	12.40 (8.05–25.22)					
Right	0.94 (0.32–2.04)	1.07 (0.39–3.60)	ns	0.64 (0.25–1.34)	1.80 (1.27–2.90)	***p*** **< 0.05**
Left	0.85 (0.28–3.57)	1.07 (0.39–3.60)	ns	0.29 (0.26–3.00)	3.10 (1.15–3.98)	***p*** **< 0.05**
Deep breath						
LH						
Right	22.10 (15.25–30.67)	25.15 (13.42–45.65	ns	21.8 (15.4–25.15)	25.5 (13.55–39.45)	ns
Left	28.35 (9.00–48.72)	21.60 (11.47–35.85)	ns	28.6 (10.7–48.65)	22.6 (14.2–35.4)	ns
HF						
Right	28.95 (16.97–51.80)	35.95 (26.52–43.62)	ns	29.1 (17.85–45.7)	35.9 (25.65–43.35)	ns
Left	24.45 (13.62–45.52)	32.10 (23.92–38.57)	ns	25.0 (14.15–43.95)	31.9 (28.3–39.65)	ns
LF/HF ratio						
Right	0.80 (0.55–1.06)	0.53 (0.34–0.99)	ns	0.86 (0.45–1.37)	1.00 (0.49–1.37)	ns
Left	0.70 (0.43–1.41)	0.87 (0.37–1.36)	ns	0.65 (0.37–1.83)	1.27 (0.70–1.83)	ns
Mental calculation						
LH						
Right	20.05 (9.56-29.3)	35.5 (16.6–44.63)	ns	19.5 (5.1–30.85)	20.9 (17.65–36.6)	ns
Left	17.45 (11.33–26.8)	24.7 (13.76–53.23)	ns	18.6 (14.85–32.5)	25.4 (19.3–47.35)	ns
HF						
Right	14.2 (8.2–24.8)	15.65 (10.5–28.03)	ns	18.8 (11.55–29.2)	14.9 (11.85–15.65)	***p*** **< 0.05**
Left	13.2 (7.07–26.52)	10.7 (7.34–23.76)	ns	14.1 (7.35–22.65)	11 (9.1–14.8)	***p*** **< 0.05**
LF/HF ratio						
Right	1.10 (0.46–3.67)	1.65 (0.90–3.57)	ns	1.04 (0.23–1.36)	3.52 (1.83–4.59)	***p*** **< 0.05**
Left	1.31 (0.45–3.62)	3.84 (0.86–4.81)	ns	1.24 (0.27–1.51)	4.48 (3.06–5.14)	***p*** **< 0.05**
Handgrip						
LH	16.3 (7.83–30.57)	25.75 (9.75–38.8)	***p*** **< 0.05**	17.1 (10.07–36.05)	27.4 (12.15–50.45)	***p*** **< 0.05**
HF	16.9 (10.5–24.48)	15.2 (5.9–25.9)	ns	18.0 (11.8–24.55)	15.5 (6.28–24.75)	ns
LF/HF ratio	1.17 (0.56–2.76)	2.72 (1.11–4.27)	***p*** **< 0.05**	1.14 (0.53–3.09)	2.12 (1,01–4.19)	***p*** **< 0.05**

Value is expressed as median (interquartile range). Significant values are bolded

*HF* high frequencies, *LF* low frequencies

With regard to the handgrip LF/HF ratio, cases showed statistically lower values than controls. Therefore, we used the controls’ handgrip LF/HF ratio interquartile range (1.11–4.27) to divide the cases into two groups: Group A (*N* = 13) with an LF/HF ratio lower or equal to 1.11 (the lower limit among the controls) was regarded to be affected by ANS dysfunction; Group B (*N* = 7) with an LF/HF ratio higher than 1.11 (similar to controls) was deemed not affected. No statistically significant differences were detected between the two groups in the ICD severity (TWRSTS and GDS), disease duration, and COMPASS-31 (Table 2).

Finally, ICD patients showed a higher score on the BDI scale than controls (*p* < 0.05; 6, 20 media score; 75%) (Table 1).

### Sub-analysis results

In the subgroup analysis of subjects aged 57 years and older, cases compared to controls showed at least one autonomic symptom on the COMPASS-31 scale (cases 86% versus controls 5%; *p* < 0.01;). No other differences were detected for the additional clinical data (Table 2).

LDF spectral analysis at rest, recording from the right (RI) and left indices (LI), showed statistical significance in lower high-frequency (HF) bands in cases compared to peer controls (median RI-HF: 11.25 versus 21.40, median LI-HF: 10.2 versus 19.00) (*p* < 0.05; 71% RI, 86% LI). In addition, significance was detected in the LF/HF ratio being lower in ICD patients than controls (median RI-LF/HF: 0.64 versus 1.80, median LI-LF/HF: 0.29 versus 3.10) (*p* < 0.05; 86% RI, 86% LI) (Table 3).

During parasympathetic activation, statistical difference was not detected. Sympathetic activations by means of mental calculation and handgrip detected a lower LF/HF ratio in both conditions (mental calculation: median RI-LF/HF 1.18 versus 3.52, median LI-LF/HF 1.24 versus 4.48, *p* < 0,05; handgrip: LF/HF 1.14 versus 2.12, *p* < 0,05) (Table 3).

## Discussion

From a clinical point of view, it is uncommon for cervical dystonia patients to complain of autonomic symptoms. Dysautonomia could be subclinical in these patients, and specific questionnaires and neurophysiological analyses are needed to investigate these symptoms. Indeed, in this study, ANS abnormalities were detected in ICD patients at the COMPASS-31 scale and the short-term spectral analysis of LDF.

Most (86%) of our ICD patients presented autonomic symptoms, mainly orthostatic intolerance and gastrointestinal manifestation, according to the COMPASS-31 scale, and we detected that the more ANS domains involved, the greater the ICD severity. This is in line with findings from Hentschel and coll. [19]. They observed orthostatic hypotension and constipation in ICD patients with depressive symptoms by administering a questionnaire modified from Low et al., addressing questions about orthostatic dizziness, cold intolerance, reduced sweating, skin discoloration, dry mouth, dry eyes, genitourinary symptoms, nausea, diarrhea, constipation, or pupillary symptoms [19]. To the best of our knowledge, the investigation of ANS symptoms using the COMPASS-31 scale in ICD patients has never been reported. The COMPASS-31 is simple, more time-efficient, nevertheless still a comprehensive tool. The COMPASS-31 scale is believed to be highly reliable in ICD patients and thus suggests its use. Indeed, the current validated Non-Motor-Dystonia Questionnaire (NMD-Q) [29] encloses only two items about autonomic symptoms, i.e., orthostatic intolerance and fatigue, which may not be sufficient to investigate the whole autonomic system.

At the neurophysiological level, the short-term spectral analysis of LDF detected in ICD patients lower expression of HF bands at rest in the subgroup analysis without remarkable LF component changes, indicating a parasympathetic dysfunction. Even though the sympathetic activations (handgrip and mental calculation) induced the normal increased response in LF components, the LF/HF ratio increased less in ICD patients than in controls (handgrip in all the patients, mental calculation in the subgroup), meaning a reduced, although still present, sympathetic response. In addition, during these tasks, the HF oscillations in patients decreased not as significantly as in controls, probably due to an abnormal parasympathetic modulation. These results might reflect an altered interaction between the two ANS components, with a reduced adaptation of the parasympathetic systems during orthosympathetic activation.

Our study confirmed the parasympathetic dysfunction demonstrated by Tiple et al. [18], who hypothesized an initially parasympathetic involvement related to its highest sensibility of damage. Their spectral analysis of heart rate variability (HRV) in a group of twenty CD patients showed lower HF oscillations at rest and a lower LF/HF ratio increase during the sympathetic activity at the tilt test [18]. Tiple’s HRV results were not modified by botulinum toxin treatment, although other studies have reported a decrease in HRV in ICD patients treated with botulinum toxin compared to peers with no history of botulinum toxin treatment [30, 31].

Contrarily, Hentschel et al. (19) demonstrated decreased HRV in ICD patients only when depression symptoms were associated with cervical dystonia. They did not find a clear relation to botulinum toxin treatment (88% on Botox therapy without depression, 84% on Botox treatment with depression). Based on these results, Hentschel concluded that ICD and depressive patients showed a disequilibrium between the two ANS components with sympathetic predominance, probably due to depression symptoms [19]. Indeed, major depression patients have higher sympathetic activity than healthy subjects [32]. The Hentschel findings are not confirmed in the results of the present study, probably for the methodological differences and the lower incidence of depression found in the present study group: 10% of present patients reported BDI > 10, compared to 30% in the Hentschel group [19].

Regarding the influence of botulinum toxin on the present results, the acute effects of Botox on the autonomic nervous system can be reasonably ruled out, enrolling ICD patients with the last dose received at least 12 weeks before the assessment; however, the cholinergic hypoactivation due to the chronic nature of treatment cannot be excluded [33].

Besides, we detected more abnormalities in the subgroup analysis, including only participants aged > 57 years and older. The aging process has been associated with structural and functional changes in autonomic nerve and ganglia, leading to sympathetic tone predominance and vagal tone reduction [28]. In the present study, cases are age- and sex-matched with controls, and participants with typical ANS illnesses of aging (hypertension and diabetes) were a priori excluded. Moreover, the patients included in the subgroup analysis did not differ statistically for the ICD severity and duration compared to patients aged < 57 years old: the ICD characteristics could not explain our ANS findings. Thus, the small number of patients and the high variability in LDF amplitude might explain this “age” bias.

Finally, the somatic cervical afferents to the brainstem regulate the ANS reflexes. During turning, the vestibular system is reported to influence the sympathetic neurons (e.g., stabilization of blood pressure) and the respiratory system (e.g., maintenance of blood oxygenation) [34]. The abnormal neck posture in CD patients might induce a disturbing brainstem afferent, changing the vestibule-sympathetic and vestibule-respiratory responses [35]. We could not find any difference in the torticollis side and ANS abnormalities, nor a correspondence between the torticollis side and the ipsilateral LDF index recording. These shreds of evidence support the global influence of neck afference on ANS responses.

The abnormal neck posture in CD patients might induce pain. Several dysautonomic manifestations (i.e., edema, hyper/hypohidrosis, skin color changes, and tachycardia) have been reported in patients affected by complex regional pain syndromes (CRPS), in whom about 25% develop fixed dystonia in the affected limb [36]. According to the study, ANS function showed a general autonomic imbalance in different percentages [37, 38]. The pathophysiology of ANS abnormalities in CRPS is still unclear; it is hypothesized that neurogenic inflammation, pathological sympatho-afferent coupling, and neuroplastic central nervous system changes contribute to general ANS symptoms [36]. In addition, CRPS might induce a dysfunction in GABAergic interneurons that, in some patients, leads to dystonia and dysautonomia [39, 40]. The role of pain in ICD on ANS abnormalities has never been explored, and its contribution to dysautonomia could not be excluded. Indeed, in ICD patients, neurotransmitters’ abnormalities may lead to several non-motor symptoms [7], and low gamma-aminobutyric acid (GABA) concentration might be responsible for ANS dysfunction. In rats, the injection of a GABA agonist into preoptic neurons caused vasoconstriction in the paw pad skin [41] and increased rat tail sympathetic nerve activity [42]. In humans, low GABA levels reduce the inhibition of the salivatory nucleus on the motor vagus nucleus, leading to abnormal vagal efference [43]. Therefore, GABA contributes to both orthosympathetic and parasympathetic activity. Microneurography, histopathological, and fMRI studies have well documented how the ANS regulation could not be interpreted within a simple orthosympathetic versus parasympathetic balance. The meticulous ANS control depends on the function considered, and ortho-parasympathetic coactivation has also been detected.

## Conclusion

This pilot study provided novel evidence of the ANS’s involvement in ICD. The homogeneity of our study cases (all patients showed a torticollis pattern) and the enrollment of selected age- and sex-matched subjects, without ANS interferences (diabetes, hypertension, medication acting on ANS), allowed for unbiased evidence of ANS. However, we are aware of the main study limitations: the small sample size, the insufficient diagnostics performed, and the potentially high inter-individual variability of the method (LDF) cannot allow for valid statements. These results are preliminary and must be confirmed by further investigations. A more analytical and functional assessment of ANS function, through an extensive battery of neurophysiological tests, in a larger untreated population of dystonic patients should be considered in ICD to better define the specific role of the adrenergic and cholinergic systems.

## Data Availability

Anonymized data can be obtained upon reasonable request from qualified researchers.
